# Fibricheck detection capabilities for atrial fibrillation (FDA–AF): a multicenter validation study

**DOI:** 10.1038/s41746-025-02059-2

**Published:** 2025-11-20

**Authors:** John Sollee, Baljash Cheema, David Slotwiner, Alexander Volodarskiy, Lien Desteghe, Christophe Buyck, Hein Heidbuchel, Stavros Stavrakis, Laurent Pison, Dieter Nuyens, Maximo Rivero-Ayerza, Hugo Van Herendael, James Thomas

**Affiliations:** 1 Department of Internal Medicine, Feinberg School of Medicine, Northwestern University, Chicago, IL USA; 2 Department of Cardiology, Feinberg School of Medicine, Northwestern University, Chicago, IL USA; 3 Bluhm Cardiovascular Institute, Center for Artificial Intelligence, Northwestern University, Chicago, IL USA; 4 Department of Cardiology, New York Presbyterian Queens, Weill Cornell Medical College, New York, NY USA; 5 Centre for Research and Innovation in Care (CRIC), Department of Nursing and Midwifery Sciences, Antwerp University, Antwerp, Belgium; 6 Department of Cardiology, Antwerp Hospital, Antwerp, Belgium; 7 Faculty of Medicine and Life Sciences, Antwerp Hospital, Antwerp, Belgium; 8 Research Group Cardiovascular Diseases, GENCOR, Antwerp University, Antwerp, Belgium; 9 University of Oklahoma Health Sciences Center, Cardiovascular Section, Oklahoma City, OK USA; 10 Clinical Electrophysiology, Department of Cardiology, Hospital Oost Limburg, Genk, Belgium

**Keywords:** Atrial fibrillation, Machine learning

## Abstract

Atrial fibrillation (AF) is the most common arrhythmia worldwide and is associated with significant morbidity and mortality. FibriCheck is a medical analysis platform that uses an end-to-end algorithm to detect AF based on photoplethysmography signals recorded on consumer smartphones. The study aimed to validate FibriCheck in a multicenter, multinational cohort of 236 subjects across ten popular smartphone devices. The 12-lead electrocardiogram was used as the reference diagnosis. FibriCheck demonstrated high overall performance: accuracy 98.5% (95% CI: 98.0–99.0%); sensitivity 96.3% (95% CI: 94.4–97.7%); specificity 99.3% (95% CI: 98.8–99.7%). Performance was not affected by smartphone device or comorbid heart failure, vascular disease, hypertension, diabetes, or stroke. Sensitivity was reduced in individuals with darker skin tones and higher BMIs, but this was mitigated by technician verification. The study confirms the high accuracy, sensitivity, and specificity of the FibriCheck algorithm in detecting AF across various smartphone models and clinical subgroups.

## Introduction

Atrial fibrillation (AF) is the most common arrhythmia worldwide, which has significantly increased in both incidence and prevalence over the last 50 years, reaching the level of a cardiovascular disease (CVD) epidemic in the 21^st^ Century^1–3^. Rising AF burden is driven by population aging^4^ and increased rates of multimorbidity such as obesity, diabetes, hypertension (HTN), and chronic stress^3^. AF is causally associated with increased risk of myocardial infarction (MI), embolic ischemic stroke, heart failure (HF), and chronic kidney disease (CKD)^5,6^. Development and progression of AF and its associated comorbidities are interdependent^7,8^. For instance, a subanalysis of the Framingham Heart Study demonstrated that 37% of patients with new AF had HF, and conversely, 57% of patients with new HF had preexisting AF^8^. The prevalence of one condition was associated with a higher incidence of the other^8^. Patients with AF also have a five times increased risk of stroke, the leading cause of chronic severe disability in the US and the fifth leading cause of death^9^. Despite medical advances, AF often remains underdiagnosed, leading to preventable complications and mortality. Because AF is typically diagnosed by an in-office 12-lead electrocardiogram (ECG), paroxysmal variants and asymptomatic cases are often missed^10^. Up to 20% of patients presenting with AF-related strokes are undiagnosed^7^, over 90% of whom meet criteria for chronic oral anticoagulation^11^. In short, there remains an ongoing need to develop clinically and economically feasible methods for early and accurate AF detection and monitoring to improve global public health.

With recent advancements in microchips, sensor technologies, and cloud computing, researchers have developed a wide variety of tools for remote healthcare monitoring. Most recent innovations in the field of CVD and beyond have focused on consumer wearables or mobile smartphone applications, many of which incorporate artificial intelligence (AI)^12,13^. Photoplethysmography (PPG) has emerged as the preferred signal modality for measuring heart rate and detecting arrhythmias, as smartphones and wearables already incorporate capable sensors^14–16^. In PPG, diodes emit light towards human tissue, and photosensors capture the reflected light. Because the intensity and pulsatility of the reflected light are a function of the propagation of arterial pressure pulses within the microvascular bed, PPG signals provide valuable real-time information about cardiovascular health, including oxygen saturation, heart rate, blood pressure, and cardiac output^17^. PPG-based AF detection algorithms typically work by extracting temporal, morphological, and/or spectral features from raw PPG signals, which are subsequently input into a classifier^15^. A recent review article identified 24 studies that incorporated PPG for AF detection, with half of these using either machine or deep learning techniques^15^.

FibriCheck [Qompium NV, Hasselt, Belgium] is a Class IIa CE and FDA-cleared medical analysis platform that utilizes an end-to-end algorithm to classify heart rhythms based on PPG signals collected through their smartphone application. Prior studies, many from the multicenter European TeleCheck-AF project, demonstrated high usability, compliance, and patient satisfaction ratings^18,19^, including in the primary care setting^20^. Small, and/or single-center studies (*N* ≤ 300) have demonstrated excellent performance in AF detection^21–24^, including in real-world conditions^25^, with the most recent validation study showing 100% sensitivity, 98.9% specificity, and 99.2% accuracy across 122 participants at a single European center^26^. Larger, multicenter validation studies in more diverse populations, including non-European participants, have not been performed. The FDA-AF study aimed to validate FibriCheck in a multicenter, multinational cohort across ten popular smartphone devices.

## Results

### Study population

**Fig. 1:**
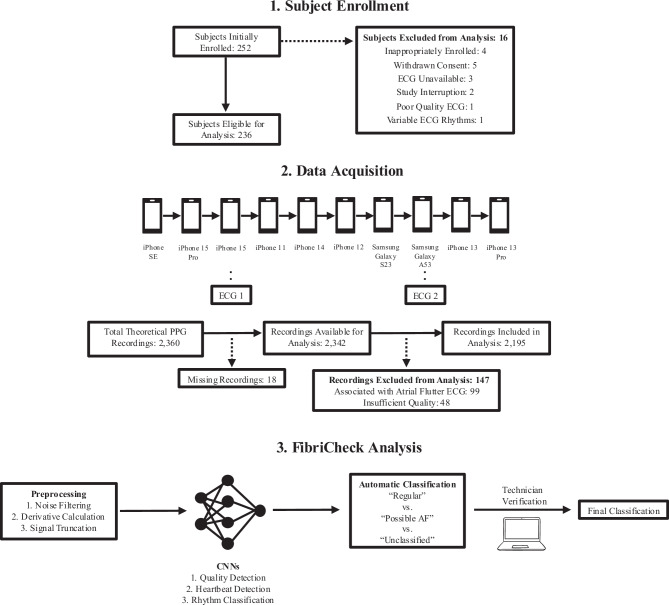
Subject enrollment, data acquisition, and FibriCheck analysis pipeline. A total of 252 subjects were initially enrolled, with 16 subsequently excluded. Participants were instructed to sequentially place their fingers on the camera of each of the 10 devices to allow for a 60-second recording. A 12-lead ECG was used as the reference standard. ECGs were performed for each participant twice during PPG recordings, once during the third smartphone recording (iPhone 15) and once during the eighth smartphone recording (Samsung Galaxy A53). Of the 2360 total theoretical recordings, 2195 were ultimately available for analysis. The recordings were preprocessed and used as input to the CNNs. The rhythm was first classified automatically and then independently reviewed by a single, blinded FibriCheck technician.

A total of 252 subjects were initially enrolled (Fig. 1). Of these, 16 (6.4%) were excluded from the study, including four who met exclusion criteria but were inappropriately enrolled, five dropouts due to withdrawn informed consent, three due to unavailability of a 12-lead ECG device during data acquisition, two due to interruption of the study by other medical examinations, one due to poor quality ECG, and one due to different rhythms being detected on the two ECG recordings for that single patient. Therefore, a total of 236 participants were eligible for analysis. Demographics and clinical information are shown in Table 1. Among the 236 participants, 157 (66.5%) were identified as having regular rhythm, and 60 (25.4%) were identified as having AF based on 12-lead ECG (reference diagnosis). Atrial flutter was identified in 10 (4.2%) participants, and the remaining nine (3.8%) participants presented with unclassified rhythms.

**Table 1:** Participant demographics and clinical characteristics

	Total Population (*n* = 236)	US population (*n* = 158)	Non-US population (*n* = 78)
Age (in years)			
Median (Q1–Q3)	65 (54–74)	65 (50–75)	66 (58.8–72)
Sex, *n* (%)			
Male	143 (60.6%)	86 (54.4%)	57 (73.1%)
Female	93 (39.4%)	72 (45.6%)	21 (26.9%)
Body Mass Index (kg/m^2^)			
Median (Q1-Q3)	28 (25-32)	29 (25-33)	27 (24.8-30)
Skin Tone, Fitzpatrick Scale			
Type I, *n* (%)	47 (19.9%)	21 (13.3%)	26 (33.3%)
Type II, *n* (%)	113 (47.9%)	71 (44.9%)	42 (53.8%)
Type III, *n* (%)	28 (11.9%)	20 (12.7%)	8 (10.3%)
Type IV, *n* (%)	16 (6.8%)	15 (9.5%)	1 (1.3%)
Type V, *n* (%)	15 (6.4%)	14 (8.9%)	1 (1.3%)
Type VI, *n* (%)	17 (7.2%)	17 (10.8%)	0 (0.0%)
Medical History			
Atrial fibrillation, *n* (%)	148 (62.7%)	86 (54.4%)	62 (79.5%)
Persistent atrial fibrillation	68 (28.8%)	31 (19.6%)	25 (32.1%)
Paroxysmal atrial fibrillation	80 (33.9%)	55 (34.8%)	37 (47.4%)
Heart failure, *n* (%)	67 (28.4%)	59 (37.3%)	8 (10.3%)
Vascular disease, *n* (%)	27 (11.4%)	22 (13.9%	5 (6.4%)
Hypertension, *n* (%)	125 (53.0%)	99 (62.7%)	26 (33.3%)
Diabetes, *n* (%)	53 (22.5%)	42 (26.6%)	11 (14.1%)
Stroke, *n* (%)	36 (15.3%)	27 (17.1%)	9 (11.5%)
COPD diagnosis, *n* (%)	15 (6.4%)	10 (6.3%)	5 (6.4%)

*US* United States, *N* number, *Q* quartile, *COPD* chronic obstructive pulmonary disease.

The theoretical maximum number of PPG recordings was 2360 (10 recordings for each participant, one recording per phone). However, 18 recordings were missing (either not collected, inadvertently deleted, or lost), leaving 2342 available for analysis. For the primary analysis, recordings associated with atrial flutter were excluded (*n* = 99). Additionally, 48 recordings were excluded for insufficient quality. Therefore, a total of 2195 recordings were available for the final primary analysis (Fig. 1). Median time from PPG recording to capture of ECG reference diagnosis was two minutes (IQR 2,3).

### Primary analysis: overall performance and performance by smartphone device

The FibriCheck algorithm demonstrated high overall accuracy and reliability in differentiating between possible and non-possible AF (Tables 2 and 3). Without technician verification, accuracy was 98.5% (95% CI: 98.0–99.0%), sensitivity 96.3% (95% CI: 94.4–97.7%), specificity 99.3% (95% CI: 98.8–99.7%), PPV 98.0% (95% CI: 96.5–98.9%), and NPV 99.8% (95% CI: 99.6–99.9%). Performance was not significantly changed with technician verification (Table 2). Performance was equivalent in US and non-US citizens (Table 2). The FibriCheck algorithm demonstrated high performance across all 10 smartphone devices, with the highest accuracy achieved in the iPhone 13 Pro (100%). There were no significant differences in accuracy among the devices. Detailed performance data for smartphone devices are shown in Table 3.

**Table 2:** FibriCheck overall performance

Overall performance	FibriCheck without verification			FibriCheck with verification		
**Group**	**All**	**US**	**Non-US**	**All**	**US**	**Non-US**
Accuracy (95% CI)	98.5% (98.0–99.0%)	98.3% (97.5–98.9%)	99.1% (98.0–99.6%)	98.8% (98.2–99.2%)	98.2% (97.4–98.8%)	99.9% (99.2–100%)
Sensitivity (95% CI)	96.3% (94.4–97.7%)	95.1% (92.2–97.2%)	98.0% (95.3–99.3%)	99.0% (97.7–99.6%)	98.2% (96.1–99.3%)	100% (98.5–100%)
Specificity (95% CI)	99.3% (98.8–99.7%	99.2% (98.5–99.6%)	99.6% (98.5–100%)	98.7% (98.0–99.2%)	98.3% (97.3–98.9%)	99.8% (98.8–100%)
Disease prevalence	26.1%	22.2%	33.8%	26.2%	22.3%	34.0%
PPV (95% CI)	98.0% (96.5–98.9%)	97.2% (94.7–98.5%)	99.2% (96.8–99.8%)	96.4% (94.7–97.6%)	94.1% (91.2–96.1%)	99.6% (97.2–99.9%)
Prevalence-adjusted PPV, 6%	90.1% (83.4–94.2%)	88.5% (80.1–93.7%)	93.8% (79.0–98.4%)	82.9% (76.1–88.2%)	78.1% (69.8–84.7%)	96.8% (81.2–99.5%)
NPV (95% CI)	98.7% (98.1–99.2%)	98.6% (97.8–99.1%)	99.0% (97.6–99.6%)	99.6% (99.2–99.8%)	99.5% (98.8–99.8%)	100% (99.2–100%)
Prevalence-adjusted NPV, 6%	99.8% (99.6–99.9%)	99.7% (99.5–99.8%)	99.5% (98.7–99.9%)	99.9% (99.9–100%)	99.9% (99.7–100%)	100% (99.2–100%)

FibriCheck performed well in differentiating possible AF from non-possible AF when all devices and patients are grouped. Performance was equivalent in US and non-US subjects.

*CI* confidence interval, *PPV* positive predictive value, *NPV* negative predictive value, *US* United States.

**Table 3:** FibriCheck performance by smartphone device

Smartphone device	Accuracy possible AF vs. non-possible AF
Overall performance (95% CI) Eligible for rhythm analysis, *n* (%)	98.5% (98.0–99.0%) 2,195/2,243 (98.2%)
iPhone SE (3rd gen) (95% CI) ΔiPhone SE (3rd gen) - overall performance Eligible for rhythm analysis, *n* (%) Time until reference diagnosis in minutes, median (Q1–Q3)	99.1% (96.8–99.9%) +0.6% 224/226 (99.1%) 6 (4,9)
iPhone 15 Pro (95% CI) ΔiPhone 15 Pro - overall performance Eligible for rhythm analysis, *n* (%) Time until reference diagnosis in minutes, median (Q1–Q3)	98.2% (95.4–99.5%) -0.3% 218/225 (96.9%) 5 (2,7)
iPhone 15 (95% CI) ΔiPhone 15 - overall performance Eligible for rhythm analysis, *n* (%) Time until reference diagnosis in minutes, median (Q1–Q3)	96.8% (93.6–98.7%) -1.7% 221/226 (97.8%) N/A, simultaneous
iPhone 11 (95% CI) ΔiPhone 11 - overall performance Eligible for rhythm analysis, *n* (%) Time until reference diagnosis in minutes, median (Q1–Q3)	98.2% (95.5–99.5%) -0.3% 224/226 (99.1%) 3 (3,4)
iPhone 14 (95% CI) ΔiPhone 14 - overall performance Eligible for rhythm analysis, *n* (%) Time until reference diagnosis in minutes, median (Q1–Q3)	99.6% (97.5–100%) +1.1% 221/225 (98.2%) 4 (3,5)
iPhone 12 (95% CI) ΔiPhone 12 - overall performance Eligible for rhythm analysis, *n* (%) Time until reference diagnosis in minutes, median (Q1–Q3)	98.7% (96.1–99.7%) +0.2% 223/225 (99.1%) 2 (1,3)
Samsung Galaxy S23 (95% CI) ΔSamsung Galaxy S23 - overall performance Eligible for rhythm analysis, *n* (%) Time until reference diagnosis in minutes, median (Q1–Q3)	97.2% (94.0–99.0%) -1.3% 213/221(96.4%) 2 (2,3)
Samsung Galaxy A54 (95% CI) ΔSamsung Galaxy A54 - overall performance Eligible for rhythm analysis, *n* (%) Time until reference diagnosis in minutes, median (Q1–Q3)	98.2% (95.4–99.5%) -0.3% 218/223 (97.8%) N/A, simultaneous
iPhone 13 (95% CI) ΔiPhone 13 - overall performance Eligible for rhythm analysis, *n* (%) Time until reference diagnosis in minutes, median (Q1–Q3)	99.5% (97.5–100%) +1.0% 217/222 (97.7%) 2 (2,3)
iPhone 13 Pro (95% CI) ΔiPhone 13 Pro - overall performance Eligible for rhythm analysis, *n* (%) Time until reference diagnosis in minutes, median (Q1–Q3)	100% (98.3–100%) +1.5% 216/224 (96.4%) 3 (3,4)

FibriCheck performed well in differentiating possible AF from non-possible AF in every device tested. “N/A, simultaneous” indicates that the ECG reference diagnosis and PPG recordings were taken simultaneously, as shown in Fig. 1.

*AF* atrial fibrillation, *CI* confidence interval, *Q* quartile, *N* number.

### Subgroup analyses

A summary of performance in each subgroup is shown in Tables 4 and 5. The FibriCheck algorithm demonstrated high accuracy across all skin tones, although sensitivity was lower in participants with darker skin tones (Fitzpatrick types V and VI). Verification by a FibriCheck technician successfully mitigated the risks associated with the lower sensitivity of 79.6% (95% CI: 65.7–89.8%) in participants with a dark skin tone compared to those with pale or medium skin tones. The FibriCheck algorithm also showed high accuracy and reliability in participants with a previous AF diagnosis. The system performed consistently well in participants with and without HF, vascular disease, HTN, diabetes, and stroke.

**Table 4:** Subgroup analysis performance without technician verification

	Possible AF vs. non-possible AF																
Subgroup	BMI ≥30	BMI <30	Pale skin	Medium skin	Dark skin	Diabetes	No diabetes	AF	No AF	HF	No HF	Stroke	No stroke	HTN	No HTN	Vascular disease	No vascular disease
Accuracy (95% CI)	97.2% (95.9–98.2%)	99.5% (98.9–99.8%)	98.7% (98.0–99.2%)	99.5% (98.3–99.9%)	96.3% (93.4–98.1%)	97.8% (96.0–98.9%)	98.8% (98.1–99.2%)	97.9% (96.9–98.6%)	–	97.5% (95.9–98.6%)	98.9% (98.3–99.4%)	97.6% (95.4–99.0%)	98.7% (98.1–99.2%)	98.1% (97.2–98.8%)	99.0% (98.2–99.5%)	100% (98.5–100%)	98.4% (97.7–98.9%)
Sensitivity (95% CI)	93.7% (89.9–96.5%)	98.2% (96.1–99.3%)	97.6% (95.7–98.8%)	100% (94.7–100%)	79.6% (65.7–89.8%)	93.6% (88.8–96.8%)	97.5% (95.5–98.8%)	96.3% (94.4–97.7%)	–	96.0% (92.3–98.3%)	96.5% (94.1–98.1%)	89.9% (81.0–95.5%)	97.4% (95.5–98.6%)	96.1% (93.6–97.8%)	96.8% (93.2–98.8%)	100% (95.1–100%)	95.8% (93.6–97.4%)
Specificity (95% CI)	98.5% (97.2–99.3%)	99.90% (99.4–100%)	99.2% (98.5–99.7%)	99.4% (98.0–99.9%)	99.6% (97.8–100%)	100% (98.9–100%)	99.2% (98.5–99.6%)	99.0% (98.0–99.6%)	99.6% (98.7–99.9%)	98.3% (96.5–99.3%)	99.7% (99.2–99.9%)	100% (98.6–100%)	99.2% (98.6–99.6%)	99.1% (98.2–99.6%)	99.5% (98.8–99.9%)	100% (97.8–100%)	99.3% (98.7–99.6%)

Performance of the automated FibriCheck algorithm in different patient subgroups before technician verification.

*AF* atrial fibrillation, *BMI* body mass index, *pale skin* Fitzpatrick Types I and II, *medium skin* Fitzpatrick types III and IV, *dark skin* Fitzpatrick types V and VI, *HF* heart failure, *HTN* hypertension, *CI* confidence interval.

**Table 5:** Subgroup analysis performance with technician verification

	Possible AF vs. non-possible AF																
Subgroup	BMI ≥30	BMI <30	Pale Skin	Medium skin	Dark skin	Diabetes	No diabetes	AF	No AF	HF	No HF	Stroke	No stroke	HTN	No HTN	Vascular disease	No vascular disease
Accuracy (95% CI)	98.2% (97.1–99.0%)	99.2% (98.5–99.6%)	98.7% (98.0–99.2%)	99.0% (97.6–99.7%)	98.6% (96.6–99.6%)	99.0% (97.6–99.7%)	98.7% (98.1–99.2%)	98.5% (97.7–99.1%)	–	97.9% (96.4–98.9%)	99.1% (98.5–99.5%)	99.4% (97.9–99.9%)	98.7% (98.0–99.1%)	99.0% (98.2–99.5%)	98.5% (97.6–99.2%)	99.2% (97.0–99.9%)	98.7% (98.1–99.2%)
Sensitivity (95% CI)	98.8% (96.4–99.7%)	99.1% (97.4–99.8%)	99.3% (98.1–99.9%)	100% (94.7–100%)	93.8% (82.8–98.7%)	98.8% (95.8–99.9%)	99.0% (97.5–99.7%)	99.0% (97.7–99.6%)	–	98.0% (95.0–99.5%)	99.5% (98.1–99.9%)	97.4% (91.0–99.7%)	99.2% (98.0–99.8%)	99.2% (97.7–99.8%)	98.4% (95.5–99.7%)	100% (95.1–100%)	98.8% (97.4–99.6%)
Specificity (95% CI)	98.0% (96.6–98.9%)	99.2% (98.4–99.7%)	98.4% (97.5–99.1%)	98.9% (97.1–99.7%)	99.6% (97.8–100%)	99.1% (97.3–99.8%)	98.6% (97.8–99.2%)	98.2% (97.0–99.0%)	99.2% (98.3–99.7%)	97.8% (95.8–99.0%)	99.0% (98.3– 99.5%)	100% (98.6–100%)	98.5% (97.6–99.0%)	98.9% (97.8–99.5%)	98.6% (97.5–99.3%)	98.8% (95.7–99.9%)	98.7% (98.0–99.2%)

Classification performance in different patient subgroups with technician verification.

*AF* atrial fibrillation, *BMI* body mass index, *pale skin* Fitzpatrick Types I and II, *medium skin* Fitzpatrick types III and IV, *dark skin* Fitzpatrick types V and VI, *HF* heart failure, *HTN* hypertension, *CI* confidence interval.

### Comparative analysis with FDA-cleared devices

Compared to the seven devices that underwent and reported clinical testing within the scope of the 510(k) clearance, FibriCheck demonstrated superior or equivalent sensitivity and specificity (Table 6).

**Table 6:** Comparative analysis with FDA-cleared devices

Device name	510(k) number	Sensitivity (95% CI)	Specificity (95% CI)
FibriCheck	K232804	96.3% (94.4–97.7%)	99.3% (98.8–99.7%)
FibriCheck	K173872	95.60% (no 95% CI reported)	96.6% (no 95% CI reported)
Coala heart monitor	K182040	97.2% (no 95% CI reported)	94.6% (no 95% CI reported)
Study watch with irregular pulse monitor	K192415	85% (79–90%)	96% (93–99%)
Halo AF detection system	K201208	93.3% (no 95% CI reported)	99.1% (no 95% CI reported)
Scan monitor	K201456	96.3% Lower bound 95% CI: 89.4%, No upper bound reported	100% Lower bound 95% CI: 96.7% No upper bound reported
Study watch with irregular pulse monitor (home) study watch with irregular pulse monitor	K213357	96.1% (92.7–98.0%)	98.1% (97.2–99.1%)
Withings scan monitor 2.0	K230812	99% (93–100%)	99% (97–100%)

FibriCheck demonstrated comparable performance to all identified devices with publicly available performance metrics.

*CI* confidence interval.

The underlined text represents data from the validation study described in the current paper.

## Discussion

The FDA-AF study validates the FibriCheck platform as a highly accurate and reliable tool for detecting AF in a diverse patient population. By demonstrating consistent performance across ten of the most common smartphone devices, the study also underscores the platform’s ease of implementation and potential as a resource-efficient method for AF detection and monitoring outside of the clinical setting.

The FibriCheck platform offers several clinical advantages over existing methods for AF detection and monitoring. Unlike traditional 12-lead ECGs, FibriCheck measurements can be performed at any time, within 60 s, and utilizing a device already owned by most patients. Patients without a formal diagnosis of AF but exhibiting symptoms may be instructed by a clinician to initiate FibriCheck readings when symptomatic. Similarly, those with paroxysmal AF may be advised to take periodic readings to assess AF burden. For select patients with paroxysmal arrhythmias who are managed with a “pill in the pocket” approach, FibriCheck could guide the self-administration of single dose antiarrhythmics (e.g., flecainide or propafenone) to terminate the arrhythmia promptly^27^. FibriCheck can also be used to monitor for arrhythmia recurrence following electrical cardioversion or ablation procedures^28,29^.

Like other patient-activated wearables, including smartwatches and handheld ECG devices, FibriCheck may miss transient or asymptomatic arrhythmias^13^. Still, unlike continuous monitoring devices such as the ZioPatch^30^, Holter monitor, or loop recorder, FibriCheck is entirely non-invasive, does not require external battery packs or chest leads, and can record and transmit unlimited readings without the need for repeat office visits or hardware exchanges^13^. Wrist-worn devices for continuous AF monitoring are being developed, such as the recently FDA-cleared Verily Study Watch^31^; however, these require the purchase of additional hardware rather than operating through a basic smartphone. In contrast, FibriCheck operates on devices already widely available, making it particularly suitable for resource-limited settings. Still, an acknowledged limitation is that only iPhone and Samsung models were included in the study. These devices are relatively more expensive compared to others available globally, such as those from Oppo, Nokia, and Optus; future studies to validate FibriCheck in these devices are encouraged.

The FibriCheck algorithm performed well across a diverse patient population, reinforcing clinical utility, particularly given high rates of multimorbidity in AF^32,33^. Several studies have demonstrated that certain comorbidities and patient characteristics, most notably obesity and skin tone, can significantly affect PPG signal quality and lead to inaccurate biophysical measurements. Skin tone is often described using the Fitzpatrick scale, which classifies skin types from I, the lightest, to VI, the darkest, based on response to ultraviolet light^34^. Monte Carlo simulations have shown that the AC/DC ratio of PPG signals, a measure of blood volume pulsatility detection, is compromised in darker skin (higher Fitzpatrick scale) due to increased light absorption by melanin^35,36^. This effect has been shown to result in signal loss in existing commercial wearables, including the Apple Watch series five and Fitbit Versa 2^36^. Obesity also affects PPG signal quality due to the effects of adipose and dermal tissue on penetration and scattering of light^37^, with effects on AC/DC signal degradation up to 40%^38^. Vascular disease and HTN have also been shown to affect PPG signals, however, likely to a lesser extent^39^.

The subgroup analysis demonstrated that FibriCheck maintains high accuracy, sensitivity, and specificity in patients with diabetes and prior stroke as well as pre-existing diagnoses of HF, HTN, and vascular disease. Sensitivity was reduced in those with darker skin tone, but this was mitigated by FibriCheck technician verification; with verification, sensitivity improved from 79.6% to 93.8%. Likewise, sensitivity was slightly reduced in individuals with a BMI of 30 or higher. This was also mitigated with technician verification, improving sensitivity from 93.7% to 98.8%. By offering technician verification, the FibriCheck platform can successfully mitigate the known effects of skin type and obesity on classification performance. This feature gives FibriCheck an advantage over other consumer platforms for mobile AF detection that do not offer human verification. Technician inter-rater agreement and external validation were not specifically assessed in this study. However, as detailed in the methods, several approaches were taken to minimize the likelihood of inter-rater variability. Future studies may investigate inter-rater agreement of visual PPG interpretation for cardiac rhythm classification.

To benchmark FibriCheck to the state-of-the-art, we performed a comparative analysis based on the performance metrics of previously cleared devices with a similar indication for use and reported clinical performance, as per the 510(k) premarket notification database. FibriCheck demonstrated comparable or equivalent performance to all identified devices reporting performance metrics^31,40^.

In conclusion, the FDA-AF study confirms the high accuracy, sensitivity, and specificity of the FibriCheck algorithm in detecting AF across various smartphone platforms and clinical subgroups. These findings support the use of FibriCheck as a reliable, low-cost, and easily accessible tool for AF detection in a diverse patient population.

## Methods

### Study design and data acquisition

The study was performed across five independent, large academic medical centers in the United States (US) and Europe: University Hospital Antwerp (UZA), Belgium; Hospital Oost-Limburg Genk (ZOL), Belgium; University of Oklahoma Health Sciences Center, Oklahoma, US; Northwestern Medicine, Chicago, US; New York Presbyterian Queens, New York, US. The institutional review boards of each institution independently approved the study, and the study followed all principles of the Declaration of Helsinki (7th edition, October 2013), per the International Council for Harmonization of Technical Requirements for Pharmaceuticals for Human Use – Good Clinical Practice (ICH-GCP) guidelines. Written informed consent was obtained for all participants. Every attempt was made to protect patient confidentiality, and participants had the right to withdraw from the study at any time.

Participants were eligible for inclusion if they met the following criteria: at least 22 years of age, capable of independently performing FibriCheck readings under observation from the study team and receiving active cardiology care either in the outpatient setting or hospitalized with or without AF. Enrollment was expected to last one month and attain 50% AF prevalence with 15–20% class IV or higher on the Fitzpatrick scale to adequately sample patients with darker skin tone (a known population where PPG signal interpretation can be less accurate)^34^. Participants were excluded if they had implantable pacemakers, cardioverter-defibrillators, or other electric devices, as such devices could interfere with natural heart rhythm; the model was not trained on such cases, and the FibriCheck platform is not intended for use in such patients. Participants were also excluded if they were unable to complete measurements independently due to physical or medical constraints, actively enrolled in other clinical trials, or pregnant or nursing women.

Data acquisition for consented patients was performed at outpatient cardiology clinics or in the hospital if patients were admitted. For each participant, the following demographic and clinical data were recorded: US or non-US citizen, BMI ≥ 30 or <30, skin type (based on the Fitzpatrick scale), presence or absence of a history of AF, HF, vascular disease, HTN, diabetes, and stroke.

The FibriCheck application with access to the FibriCheck cloud and FibriCheck algorithm (v1.5.2) was pre-installed on ten different iOS (*n* = 8) and Android (*n* = 2) devices. Participants were instructed to sequentially place their fingers on the camera of each of the ten devices to allow for a 60-s PPG reading as outlined in Fig. 1. The number of recording attempts per smartphone device was determined by the FibriCheck quality analysis, described below. If a PPG recording was deemed to have insufficient quality according to the FibriCheck algorithm, participants were instructed to repeat the recording until adequate quality was achieved, with a maximum of three attempts permitted per smartphone. At the end of each recording, the PPG data was automatically transmitted to the FibriCheck Cloud for processing and analysis by the FibriCheck algorithm.

A 12-lead ECG was used as the reference standard. ECGs were performed for each participant twice during PPG recordings, once during the third smartphone PPG recording (Apple iPhone 15) and once during the eighth smartphone PPG recording (Samsung Galaxy A53) (Fig. 1). Each ECG was evaluated by at least two board-certified and independent cardiac electrophysiologists and labeled as “regular,” “AF,” “atrial flutter,” or “unclassified” (not one of the other rhythms). If there were discrepancies in the findings of the two experts, then a third expert was consulted, and the majority decision decided the reference diagnosis. In the case of disagreement among all three experts or if the ECG was deemed unreadable (poor quality), the data were excluded from the analysis. If the two ECGs for a single patient were considered to be different rhythms, then the data were also excluded.

### FibriCheck platform algorithm

The FibriCheck platform uses an end-to-end algorithm incorporating three convolutional neural networks (CNNs): (1) *quality detection*, (2) *heartbeat detection*, and (3) *rhythm classification*.

To initiate data collection, users place their finger on the smartphone camera lens. Once the presence of a finger is confirmed using a dedicated detection algorithm, a video is recorded in YUV color format for one minute. After recording, the video is converted to the RGB color format, where the RGB components are treated as potential PPG signals. Each recording is 60 s. The RGB signals then undergo a series of preprocessing steps, which include noise filtering, derivative calculation, normalization, and signal truncation. The RGB color channels contain the raw PPG time series signal information; as such, the preprocessing steps aim to enhance the quality of the encapsulated PPG signals by reducing the influence of noise and/or artifacts.

The preprocessed PPG time series signals are used as input to the three CNNs. The (1) *quality detection* CNN indicates if specific segments within the PPG are too noisy for further analysis. If the model determines that more than 30 s of the PPG signal is too noisy or fails to meet quality standards, the measurement is flagged as “insufficient quality,” and no further clinical analysis is performed. Sufficient quality signals are passed to the (2) *heartbeat detection* CNN, which indicates the location of heartbeats in the preprocessed PPG signal and constructs a PPG-based tachogram and average heart rate measurement over the one-minute measurement. Both the (1) *quality detection* and (2) *heartbeat detection* models are trained on beat-to-beat annotated internal PPG datasets consisting of tens of thousands of synchronized PPG-ECG data samples.

When the signal quality meets the required criteria and the heart rate falls within the validated range, the platform proceeds to the (3) *rhythm classification* CNN. This model has been trained on a diverse dataset of over one million rhythm-annotated measurements, encompassing various heart rhythm disorders, including AF and atrial flutter, amongst others. The algorithm then classifies the heart rhythm based on the PPG signals as “regular,” “possible AF,” or “unclassified” (i.e., not one of the other rhythms).

### Technician verification

In addition to fully automated classification by the *rhythm classification* CNN as above, each PPG recording of sufficient quality is independently reviewed by a single, blinded FibriCheck technician. Verification occurs within 48 h and is performed on a per-PPG measurement basis: each measurement is first analyzed by the algorithm and then automatically queued for human review. The technician is blinded to all patient demographic and clinical data as well as the classification output of the automated algorithm, negating the opportunity to introduce bias. By visually reviewing the PPG recordings, the technician classifies the rhythm as “regular,” “possible AF,” or “unclassified” based on strict and precise criteria extrapolated from the peer-reviewed practical guidance on signal interpretation and clinical scenarios from TeleCheck-AF^41^. To further minimize the chance of human rater variability, technicians are extensively trained and follow a standardized and structured stepwise approach^41^. It has been demonstrated that trained readers generally perform well in classifying cardiac rhythm based on raw PPG signals alone^42^.

Criteria for “regular” rhythm include equal intervals between peaks in the raw PPG signal, sporadic irregularity, a single line or wave-like pattern in the tachogram, and a dense or ellipse-shaped cluster in the Poincaré plot. Criteria for “possible AF” are irregularly varying intervals between the peaks in the PPG tracing, randomly distributed points on the tachogram, and the absence of a distinct cluster of points on the Poincaré plot. If the recording does not meet criteria for either “regular” rhythm or “possible AF,” then it is labeled “unclassified.” In cases of discrepancies between automated and human classifications, the human classification takes precedence, and the final classification is adjusted accordingly.

### Statistical analysis and outcome measures

Sample size calculations were performed before subject recruitment. Based on European post-market surveillance data, an accuracy of 95.8% (95% CI: 93.76–97.32%) can be expected in detecting AF. For the analysis, the reference value (p0) was set to the lower bound of the accuracy CI, which was 93.8%. The expected accuracy of the FibriCheck system was set at *p* = 0.990 based on European data. Using the exact Clopper & Pearson method and aiming for a significance level (alpha) of 0.025 and a power (1-beta) of 0.8, the calculated sample size required for this comparison was determined to be 114 recordings of sufficient signal quality. To allow for potential subanalyses, the targeted number of participants was set at 250.

The primary analysis assessed the ability of FibriCheck to differentiate “possible AF” from “non-possible AF.” FibriCheck classifications of “regular” and “unclassified” were grouped as “non-possible AF.” As the ECG reference diagnoses were classified as “regular,” “AF,” or “unclassified,” “regular” and “unclassified” were grouped as “non-AF.” Therefore, if FibriCheck classified a PPG recording as “possible AF,” and the associated ECG reference diagnosis was “AF,” then this was deemed a correct classification. Conversely, if FibriCheck classified a PPG recording as “possible AF,” and the associated ECG reference diagnosis was either “regular” or “unclassified” (“non-AF”), then this was deemed an incorrect classification. If FibriCheck classified a PPG recording as either “regular” or “unclassified” (“non-possible AF”), and the associated ECG reference diagnosis was “AF,” then this was also deemed an incorrect classification. Note that per FDA regulations, PPG recordings can only diagnose “possible AF,” whereas an electrophysiologist reading an ECG is the diagnostic gold standard and thus can diagnose “AF.”

Performance was first assessed across all devices and participants collectively. Performance was also stratified by US and non-US citizens. Subgroup analyses were conducted to determine performance in individual devices across different skin tones, in individuals with a BMI above and below 30, and in those with and without a previous diagnosis of AF, HF, HTN, diabetes, vascular disease, or stroke. For the analysis in patients with different skin tones, three groups were defined based on the Fitzpatrick scale: “pale” (types I and II), “medium” (types III and IV), and “dark” (types V and VI). This grouping protocol is well recognized in the literature.

Continuous variables were presented as means with standard deviations (SD) or medians with interquartile ranges (IQR). Categorical values were reported as counts and percentages. The performance of FibriCheck was evaluated by calculating the accuracy, sensitivity, specificity, positive predictive value (PPV), and negative predictive value (NPV), along with their corresponding 95% confidence intervals (CIs). Prevalence-adjusted PPV and NPV were also calculated assuming an AF prevalence of 6%. Data was analyzed using MedCalc Software Ltd.’s Diagnostic Test Evaluation Calculator (Version 22.016).

### Comparative analysis with FDA-cleared devices

To benchmark FibriCheck in comparison to the generally acknowledged state-of-the-art, we searched the publicly available 510(k) premarket notification database (https://www.accessdata.fda.gov). Since January 1, 2015, 57 devices were cleared within the DXH product code. Among these, 22 devices had a similar indication for use, specifically focusing on self-testing by patients diagnosed with or at risk of AF. Out of these 22 devices, seven underwent and reported clinical testing within the scope of the 510(k) clearance and were included in the final analysis.

## Data Availability

The datasets generated and/or analyzed during the current study are not publicly available due to intellectual property concerns. However, they are available from the corresponding author upon reasonable request, provided permission is obtained from FibriCheck. The code underlying the FibriCheck algorithm is proprietary and therefore not publicly available due to intellectual property concerns. The code used for data analysis is available from the corresponding author upon reasonable request and with permission from FibriCheck.
